# Prediction model for short-term mortality after palliative radiotherapy for patients having advanced cancer: a cohort study from routine electronic medical data

**DOI:** 10.1038/s41598-020-62826-x

**Published:** 2020-04-01

**Authors:** Shing Fung Lee, Hollis Luk, Aray Wong, Chuk Kwan Ng, Frank Chi Sing Wong, Miguel Angel Luque-Fernandez

**Affiliations:** 1 Department of Clinical Oncology, Tuen Mun Hospital, New Territories West Cluster, Hospital Authority, Hong Kong, Hong Kong; 2 Department of Non-Communicable Disease and Cancer Epidemiology, Institute de Investigacion Biosanitaria de Granada (ibs.GRANADA), University of Granada, Granada, Spain; 3 Department of Non-Communicable Disease Epidemiology, London School of Hygiene and Tropical Medicine, London, UK

**Keywords:** Oncology, Outcomes research, Cancer

## Abstract

We developed a predictive score system for 30-day mortality after palliative radiotherapy by using predictors from routine electronic medical record. Patients with metastatic cancer receiving first course palliative radiotherapy from 1 July, 2007 to 31 December, 2017 were identified. 30-day mortality odds ratios and probabilities of the death predictive score were obtained using multivariable logistic regression model. Overall, 5,795 patients participated. Median follow-up was 39.6 months (range, 24.5–69.3) for all surviving patients. 5,290 patients died over a median 110 days, of whom 995 (17.2%) died within 30 days of radiotherapy commencement. The most important mortality predictors were primary lung cancer (odds ratio: 1.73, 95% confidence interval: 1.47–2.04) and log peripheral blood neutrophil lymphocyte ratio (odds ratio: 1.71, 95% confidence interval: 1.52–1.92). The developed predictive scoring system had 10 predictor variables and 20 points. The cross-validated area under curve was 0.81 (95% confidence interval: 0.79–0.82). The calibration suggested a reasonably good fit for the model (likelihood-ratio statistic: 2.81, P = 0.094), providing an accurate prediction for almost all 30-day mortality probabilities. The predictive scoring system accurately predicted 30-day mortality among patients with stage IV cancer. Oncologists may use this to tailor palliative therapy for patients.

## Introduction

Many patients with metastatic cancer receive oncological treatment, and radiotherapy (RT) is an important component of palliative treatment^1^. RT can be an effective tool for palliation of symptoms arising from cancer, including pain from bone metastases or neurological compromise from brain or spinal metastases with cord or nerve root compression. The aim of palliative RT is to alleviate symptoms and improve quality of life. Evidence has shown that palliative RT was received by approximately 10% of patients who died of cancer near their end of life^2,3^. In one population-based study that included 15,287 patients who received RT in the last month of life, 17.8% received more than 10 days of treatment^4^. This finding corroborates with a German study which showed 50% of patients spent more than 60% of remaining 30 days of life receiving RT^4^.

RT can be delivered via different dosing regimens (e.g., single fraction on one day versus multiple fractions for weeks)^5–9^. The use of multi-fractionation (split up the total dose into small fractions) is often perceived to be associated with less long-term complications and need for retreatment^4^. While larger fraction size by single-fractionation theoretically has an increased risk of late-onset radiation toxicity. Beside radiobiological consideration, medical training and experience, departmental policies, and insurance reimbursement all influence the decision on the dose-fractionation regimens. Use of hypofractionation or single fractionation is associated with a perceived poor prognosis by the oncologist. Protracted courses of RT can become considerable demand and burden on terminal cancer patients^7^. Their symptoms can be aggravated by transportation to the RT facility and repeated positioning in the treatment suite. It is also costly to the healthcare system but might also preclude the trigger for end-of-life measures for this group of patients.

As highlighted in the statement by the American Society of Clinical Oncology in 2011, transition from focusing on cancer-directed therapy to palliative care often occurs within days of death^10^. This is possibly related to the poor accuracy of clinicians at predicting prognosis and survival of patients with advanced malignancies^11–13^. An accurate and practical short-term mortality prediction score for patients with metastatic cancer receiving palliative RT can assist clinicians in tailoring palliative RT use and for delivering appropriate dose-fractionations according to the expected short-term risk of death. Furthermore, an earlier and more thorough assessment of patient management options, goals, and preferences to facilitate personalized palliative care along the disease trajectory will be possible. When a cure is not possible, the goals of treatment change appropriately from prolonging life to controlling symptoms and improving quality of life. However, evidence has shown that clinician estimates of survival tend to be optimistic and poorly reproducible^11^. Prognosis overestimates may have contributed to mismatched fractionation schedules, and lots of patients needing to discontinue therapy.

The determination of prognosis and life expectancy is critical to the care of patients with advanced cancer. Prognostic factors and predictive tools have been explored and developed to improve the estimation of life expectancy over the clinician’s estimation. However, most of these studies are based on small study samples and hospital settings with short follow-up and survival times (in terms of weeks) or based on single tumor sites and are not specific to patients referred for RT^14–17^. Some studied the prognostic factors for survival time after palliative RT but did not develop a predictive model^3,18^. Palliative RT rarely affect survival time but often improves quality of life^19,20^. To improve palliative RT delivery and resource allocation optimization, we conducted a cohort study using routinely collected electronic data from patients with metastatic cancer receiving their first course of palliative RT. We studied the factors associated with short-term mortality and developed a predictive score model for 30-day mortality.

## Methods

### Data source

We retrieved data for our patient cohort on April 1, 2019. All RT episodes were identified using the MOSAIQ information system^42^, which archives and integrates RT planning and treatment details. These data were then linked to the entries maintained by the Clinical Data Analysis and Reporting System, which is an electronic medical record (EMR) database operated by the Hospital Authority of Hong Kong. The International Classification of Diseases, ninth revision (ICD-9), was used for disease coding. We followed the transparent reporting of a multivariable prediction model for individual prognosis or diagnosis (TRIPOD) guideline for model development and validation^43,44^. The Research Ethics Committee, New Territories West Cluster, Hospital Authority, Hong Kong approved the study on October 1, 2018, and waived patient consent requirement (reference no: NTWC/REC/18093). The research was conducted in accordance with the 1964 Declaration of Helsinki and its later amendments.

### Patients, data, and settings

We included patients with metastatic cancer who received palliative RT at Tuen Mun Hospital, Hong Kong, between July 1, 2007 and December 31, 2017, and had not received palliative radiotherapy at the center before July 1, 2007. The definition of first course palliative RT fraction was based on a combination of the receipt of an identified palliative dose-fractionation and the treating oncologist’s indication; a RT course is defined as one or more fractions of external beam RT, delivered to a defined area.

### Short-term mortality predictive risk factors

To transform raw EMR data into variables usable in a prediction model, we first collected all data from the 180- to 365-day period (depending on particular variables), ending the day before palliative RT initiation (we did not exclude patients based on absence of data during the period). Raw data were aggregated into potential predictors in the following categories: demographics, prescribed medications, comorbidities and other grouped ICD-9 diagnoses, surgical procedures, health care resource use, and laboratory results. No data on the first course palliative RT itself (e.g., dose-fractionation and techniques) were used in the predictive model. More precise information on the variables used as short-term mortality predictors are provided in Appendix 1 and Supplementary Table 3.

### Outcomes

Our primary outcome was 30-day overall mortality, which was calculated from the start of the first course palliative RT until death or when censored (April 1, 2019). The start date of RT was used because it was closer to the date when the clinical decision to treat was made than that of the end of treatment and provides a uniform time point across all fractionation regimens.

### Model selection, performance, and scoring

We used multivariable logistic regression models to evaluate the predictive performance of the primary outcome, 30-day mortality^45,46^. The model’s predictor functions were pre-specified a priori based on subject matter knowledge (Table 2). We assumed a pattern of randomness and created one imputed dataset using a fully conditional specification based on a multivariate normal distribution^47^. Different combinations of the 13 covariates were chosen for the regression models (Table 2). The 13 covariates were age, sex, Royal College of Surgeons modified comorbidity score^48^, log peripheral white blood cell count, log peripheral blood neutrophil lymphocyte ratio (NLR), log plasma urea, log serum bilirubin, serum albumin, lactate dehydrogenase (LDH), red cell distribution, attendance to emergency room, sites receiving palliative RT, and primary lung cancer.

Data-adaptive methods based on cross-validation and mean absolute error for predictions (MAE) were used to evaluate the predictive performance of different model specifications. We used ten-fold cross-validation to reduce the risk of overfitting the final model to the training set^49^. The cross-validation procedure involved fitting a candidate model for the primary outcome, using data from nine of the ten blocks (the “derivation set”), and evaluating its performance in the held-out block (the “validation set”). We repeated this process ten times, each time using a different block as the validation set, and then averaged the performance over the ten validation sets.

As the overall performance metric, we used the MAE, which measures the average of the difference between predicted and observed outcome in the test, i.e., the average prediction error^50^. This represents the closeness of the prediction to the eventual outcomes. Our measure of model discrimination was the cross-validated areas under the receiver operating characteristic (ROC) curves^51,52^. An ROC curve is a plot of the sensitivity of a model (the vertical axis) vs 1 minus the specificity (the horizontal axis) for all possible cut-off values that might be used to classify patients predicted to have 30-day mortality compared with patients who will not die within 30 days^51^. Given any 2 random patients, one died within 30 days and one did not, the probability that the model will correctly classify the patient with the outcome as higher risk is equal to the area under the ROC curve (AUC)^53^. We calculated 95% confidence intervals (CIs) of the AUC following the method of DeLong *et al*.^54^. We evaluated the model calibration by observing the agreement between observed outcomes and predictions^55^. We used a graphical assessment of calibration, with predictions on the x-axis and the observed outcome on the y-axis. We performed a sensitivity analysis to evaluate the robustness of model performance by testing different model specifications.

Finally, we produced a point score system from the best model we developed. In the system, points were assigned based on the predictor values for a patient; the total scores correspond to the risks of the 30-day overall mortality^56^. The steps to develop the point score system have been summarized in the Appendix 2. For each point score we summarized the positive predictive value (PPV) and negative predictive value (NPV) which respectively represented the probability that the disease is present given a positive test result and that the disease is absent given a negative test result^57^.

### Statistical analysis

Descriptive analyses were conducted to describe the cohort of patients receiving first course palliative RT. We used frequencies and proportions for categorical variables and means with standard deviations (when normally distributed) or medians with interquartile ranges (when not normally distributed) for continuous variables. To describe the association between patient factors and an increased or decreased short-term mortality, we reported odds ratios (OR) from univariable and multivariable logistic regressions with their respective 95% CI.

The analysis was performed using Stata v.15.1 (StataCorp LLC, College Station, Texas, USA) and R v. 3.6.1 (R Foundation for Statistical Computing, Vienna, Austria)^58,59^.

## Results

### Description of the cohort

We identified 5,795 patients who commenced palliative RT between July 1, 2007 and December 31, 2017. Patient characteristics are summarized in Table 1. The median age was 64 (interquartile range: 55–75) years; 61.8% were male. Patients with lung cancer (39.7%) constituted the highest proportion of the cohort. In all, 55.1%, 29.2%, and 15.7% were classified as having score 0, 1, and ≥2, according to the Royal College of Surgeons modified Charlson score, respectively. A total of 5,291 patients died during the follow-up period (median follow-up 3 months), of which 995 patients (17.2%) died within 30 days from the start of RT. Data were complete except for those on albumin, peripheral blood cell counts, urea, bilirubin, and LDH, which were imputed^21^.

**Table 1:** Patient characteristics for model derivation.

Variables	Complete cohort (n = 5,795)
**Age (years)**	
Median (interquartile range)	64 (55–75)
<55 years [cases (%)]	1,310 (22.6)
55–64 years [cases (%)]	1,680 (29.0)
65–74 years [cases (%)]	1,354 (23.4)
≥75 years [cases (%)]	1,451 (25.0)
**Sex [cases (%)]**	
Male	3,582 (61.8)
Female	2,213 (38.2)
**Royal College of Surgeons modified Charlson score [cases (%)]**	
0 comorbidity	3,194 (55.1)
1 comorbidity	1,691 (29.2)
≥2 comorbidities	910 (15.7)
**Primary cancer sites [cases (%)]**	
Head and neck cancers	318 (5.5)
Upper GI cancers	799 (13.8)
Lower GI cancers	588 (10.1)
Lung and thoracic cancers	2,299 (39.7)
Breast cancers	579 (10.0)
Soft tissue and skin cancers	76 (1.3)
Genitourinary cancers	693 (12.0)
Hematological cancers	121 (2.1)
CNS cancers	64 (1.1)
Other cancers	258 (4.5)
**Radiotherapy fractionations [cases (%)]**	
1	900 (15.5)
2–4	418 (7.2)
5	2,611 (45.1)
6–9	680 (11.7)
≥10	1,186 (20.5)
**Preceding radical/adjuvant radiotherapy [cases (%)]**	
Yes	525 (9.1)
No	5270 (91.0)
**Treatment-free interval [cases (%)]**	
<6 months	109 (20.8)
6–12 months	98 (18.7)
>12–24 months	136 (25.9)
>24 months	182 (34.7)

Abbreviation: CNS, central nervous system.

### Thirty-day mortality and model performance

Of the 5,795 patients receiving their first course palliative RT, 995 (17.2%) died within 30 days. Model 2 was chosen as the best performing model among candidate models 1–4 from the regression analyses (Table 2). The most important predictors of short-term mortality were primary lung cancer (OR: 1.73, 95% CI: 1.47–2.04), log peripheral blood NLR (OR: 1.71, 95% CI 1.52–1.92), and log plasma urea (OR: 1.55, 95% CI: 1.32–1.82).

**Table 2:** Comparison of different model specifications.

Variables	Univariable OR (95% CI)	Model 1: Adjusted OR (95% CI)	Model 2: Adjusted OR (95% CI)	Model 3: Adjusted OR (95% CI)	Model 4: Adjusted OR (95% CI)
Age, per one-year increase	1.01 (1.01–1.02)	—	—	1.00 (1.00–1.01)	1.00 (1.00–1.01)
Sex, male vs. female	1.45 (1.25–1.68)	—	—	1.13 (0.95–1.34)	1.13 (0.96–1.35)
RCS comorbidity score	0.95 (0.87–1.05)	—	—	—	0.87 (0.78–0.97)
Log peripheral white blood cell count (10^9^ cells/L)	4.64 (3.98–5.40)	1.52 (1.25–1.84)	1.40 (1.14–1.70)	1.40 (1.15–1.71)	1.39 (1.14–1.70)
Log peripheral neutrophil lymphocyte ratio	2.84 (2.60–3.10)	1.76 (1.57–1.97)	1.71 (1.52–1.92)	1.72 (1.53–1.93)	1.72 (1.53–1.93)
Log plasma urea (mmol/L)	2.06 (1.78–2.39)	1.53 (1.30–1.78)	1.55 (1.32–1.82)	1.49 (1.26–1.76)	1.53 (1.29–1.81)
Log serum bilirubin (µmol/L)	1.75 (1.58–1.94)	1.40 (1.25–1.57)	1.49 (1.33–1.67)	1.48 (1.32–1.66)	1.49 (1.32–1.68)
Serum albumin (g/dL)	0.87 (0.85–0.88)	0.91 (0.89–0.92)	0.90 (0.89–0.91)	0.90 (0.89–0.91)	0.90 (0.89–0.91)
Lactate dehydrogenase (IU/L)	1.00 (1.00–1.00)	1.00 (1.00–1.00)	1.00 (1.00–1.00)	1.00 (1.00–1.00)	1.00 (1.00–1.00)
Red cell distribution, per % increase	1.08 (1.06–1.11)	1.23 (1.03–1.46)	1.30 (1.09–1.56)	1.31 (1.09–1.57)	1.31 (1.09–1.57)
Attendance to emergency room	1.70 (1.54–1.88)	—	1.43 (1.28–1.60)	1.43 (1.28–1.59)	1.44 (1.29–1.61)
Sites receiving palliative radiotherapy, whole brain or spinal RT vs otherwise	1.16 (1.01–1.34)	—	1.45 (1.23–1.71)	1.46 (1.24–1.73)	1.45 (1.23–1.72)
Primary lung cancer, yes vs no	1.41 (1.20–1.62)	—	1.73 (1.47–2.04)	1.69 (1.43–2.00)	1.67 (1.42–1.98)
**Model Performance**		**Model 1**	**Model 2**	**Model 3**	**Model 4**
Cross-validated AUCs (10-fold)		0.80 (0.78–0.81)	0.81 (0.79–0.82)	0.81 (0.79–0.82)	0.81 (0.79–0.82)
MAEs (10-fold)		0.22865794	0.22170616	0.22357565	0.22318065
Log-likelihood		-2139.5312	-2087.5888	-2086.2183	-2082.9179
P-value of likelihood ratio test (1 vs. 2)		<0.0001			
P-value of likelihood ratio test (1 vs. 3)		<0.0001			
P-value of likelihood ratio test (1 vs. 4)		<0.0001			
P-value of likelihood ratio test (2 vs. 3)			0.2540		
P-value of likelihood ratio test (2 vs. 4)			0.0251		
P-value of likelihood ratio test (3 vs. 4)				0.0102	

AUC: area under curve; CI: confidence interval; MAEs: Mean absolute error for predictions (observed – predicted). OR: odds ratio; RCS: Royal College of Surgeons.

Missing values needing imputation: log peripheral white blood cell count n (%) = 243 (4.2%), log peripheral neutrophil lymphocyte ratio n (%) = 378 (6.5%), log plasma urea n (%) = 177 (3.1%), log serum bilirubin n (%) = 132 (2.3%), serum albumin n (%) = 198 (3.4%), lactate dehydrogenase n (%) = 2,730 (47.1%), red cell distribution n (%) = 243 (4.2%).

**Figure 1:**
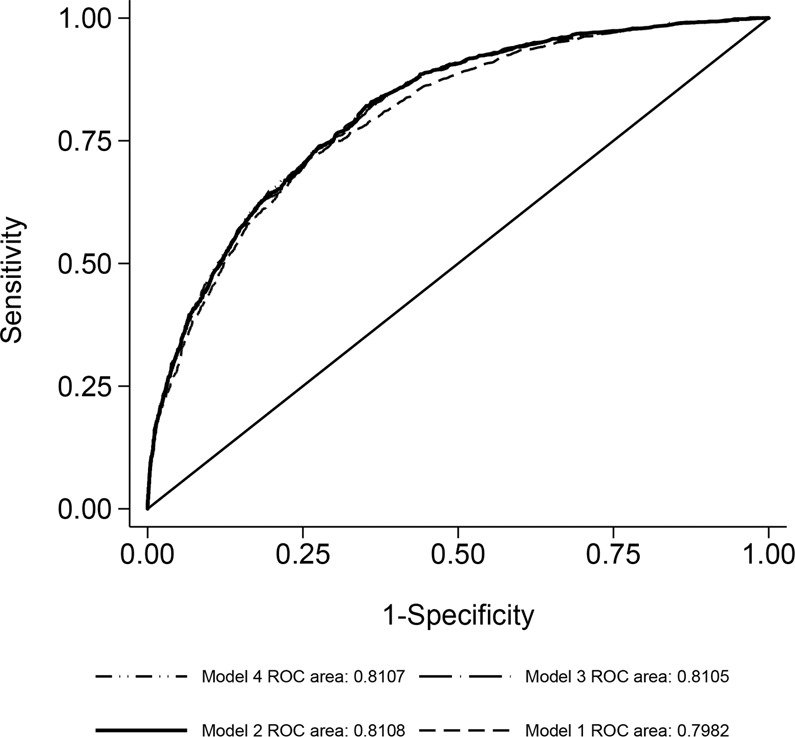
Model discriminations from the four candidate models by the receiver-operating characteristics (ROC) curves, as shown by the respective areas under curve. (created using Stata v.15.1, https://www.stata.com/).

**Figure 2:**
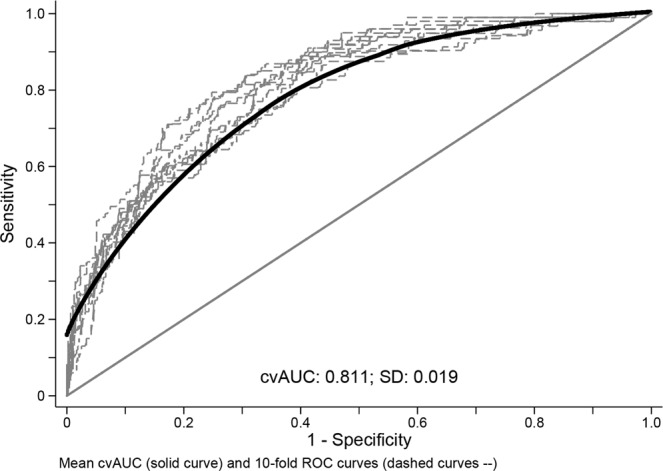
10-fold cv-ROC curves for 30-day mortality prediction from the best candidate model (model 2), which has a cv-area under curve of 0.811. SD: standard deviation; cv-ROC: cross-validated receiver-operating characteristics. (created using Stata v.15.1, https://www.stata.com/).

Figure 1 shows good model discriminations from the candidate models by the ROC curves. Figure 2 shows the 10-fold cross-validated receiver-operating characteristics (cv-ROC) curve for 30-day mortality prediction from the best model (model 2 in Table 2). Model 2 showed the highest discrimination, i.e., its predictive accuracy was good, with a cross-validated-area under curve (cvAUC) of 0.81 (95% CI: 0.79–0.82) (Figs. 1,2).

Tables 3 and 4 show the point score and average predicted probabilities of 30-day mortality based on model 2, respectively. For ease of interpretation, values of the predictors in log-scale were back-transformed to their original scale. A point score cut-off value of 6 (positive predictive value: 33.0%; negative predictive value: 94.2%; sensitivity: 80.3%; specificity: 66.2%) showed the greatest Youden’s index (46.5), corresponding to maximum joint sensitivity and specificity on the ROC curve.

**Table 3:** Points score system for probability of 30-day mortality for patients with metastatic cancer receiving first course palliative radiotherapy based on model derived.

Predictors	Transformed values	Points
**Log peripheral white blood cell count**	**(10**^**9**^ **cells/L)**	
**0–1.9**	**0–6.7**	0
>1.9–2.3	>6.7–10.0	1
>2.3–2.9	>10.0–18.2	1
**Log peripheral blood neutrophil-lymphocyte ratio**		
**-2.3–1.1**	**0.1–3.0**	0
>1.1–1.6	>3.0–5.0	2
>1.6–2.3	>5.0–10.0	3
>2.3–3.3	>10.0–27.1	3
**Log plasma urea**	**(mmol/l)**	
**-0.2–1.4**	**0.8–4.1**	0
>1.4–1.8	>4.1–6.0	1
>1.8–2.4	>6.0–11.0	1
**Log serum bilirubin**	**(µmol/L)**	
**0–3.2**	**0–25**	0
>3.2–4.1	>25–60	2
>4.1–6.4	>60–602	3
**Serum albumin (g/dL)**		
11–33	—	5
34–38		2
**39–52**		0
**Lactate dehydrogenase (IU/L)**		
**74–347**	—	0
348–798		0
799–1,774		1
**Red cell distribution**		
**11.2–13.3**	—	0
>13.3–14.0		1
>14.0–14.7		1
>14.–15.8		1
>15.8–21.8		3
**Attendance to emergency room**		
**0 time**	—	0
1 time		1
≥2 times		1
**Sites receiving palliative RT**		
Whole brain or spinal RT	—	1
**Otherwise**		0
**Primary lung cancer**		
Yes	—	1
No		0

**Table 4:** Probabilities of the outcome (30-day mortality) that correspond to the points total.

Points total	Probabilities of 30-day mortality	Sensitivity in % (95% CI)	Specificity in % (95% CI)	Positive predictive value in % (95% CI)	Negative predictive value in % (95% CI)	Youden Index
0	0.006308607	100.0 (99.6–100.0)	0.3 (0.2–0.5)	17.2 (16.3–18.2)	100.0 (80.6–100.0)	0.3
1	0.010683368	100.0 (99.6–100.0)	2.0 (1.6–2.4)	17.5 (16.5–18.5)	100.0 (96.1–100.0)	2.0
2	0.018036781	99.5 (98.8–99.8)	6.3 (5.7–7.1)	18.0 (17.1–19.1)	98.4 (96.3–99.3)	5.8
3	0.030296585	98.7 (97.8–99.2)	16.3 (15.3–17.3)	19.6 (18.6–20.8)	98.4 (97.2–99.0)	15.0
4	0.050461291	96.6 (95.3–97.5)	31.4 (30.1–32.8)	22.6 (21.4–23.9)	97.8 (96.9–98.4)	28.0
5	0.082899786	92.0 (90.1–93.5)	49.3 (47.9–50.7)	27.3 (25.9–28.9)	96.7 (96.0–97.4)	41.3
6	0.133264410	80.3 (77.7–82.7)	66.2 (64.8–67.5)	33.0 (31.1–34.9)	94.2 (93.3–94.9)	46.5
7	0.207310609	65.0 (62.0–67.9)	79.5 (78.4–80.7)	39.7 (37.4–42.1)	91.6 (90.8–92.4)	44.5
8	0.307884580	49.3 (46.2–52.4)	88.9 (88.0–89.7)	47.9 (44.9–51.0)	89.4 (88.5–90.3)	39.6
9	0.430737634	33.7 (30.8–36.7)	94.9 (94.3–95.5)	58.0 (53.9–61.9)	87.3 (86.4–88.2)	28.6
10	0.562753168	20.3 (17.9–22.9)	98.0 (97.6–98.4)	68.0 (62.5–73.1)	85.6 (84.6–86.5)	18.3
11	0.686440139	11.5 (9.6–13.6)	99.4 (99.1–99.5)	78.6 (71.3–84.5)	84.4 (83.4–85.3)	10.9
12	0.788300949	4.8 (3.7–6.3)	99.8 (99.6–99.9)	81.4 (69.6–89.3)	83.5 (82.5–84.4)	4.6
13	0.863644966	1.4 (0.8–2.3)	100.0 (99.8–100.0)	87.5 (64.0–96.5)	83.0 (82.0–84.0)	1.4
14	0.915063165	0.5 (0.2–1.2)	100.0 (99.9–100.0)	100.0 (56.6–100.0)	82.9 (81.9–83.8)	0.5
15	0.948253778	0.1 (0.0–0.6)	100.0 (99.9–100.0)	100.0 (20.7–100.0)	82.8 (81.9–83.8)	0.1
16	0.968915142	0.1 (0.0–0.6)	100.0 (99.9–100.0)	100.0 (20.7–100.0)	82.8 (81.9–83.8)	0.1
17	0.981487838	0.0 (0.0–0.4)	100.0 (99.9–100.0)	*	82.8 (81.8–83.8)	0.0
18	0.989032894	0.0 (0.0–0.4)	100.0 (99.9–100.0)	*	82.8 (81.8–83.8)	0.0
19	0.993523083	0.0 (0.0–0.4)	100.0 (99.9–100.0)	*	82.8 (81.8–83.8)	
20	0.996181981	0.0 (0.0–0.4)	100.0 (99.9–100.0)	*	82.8 (81.8–83.8)	

*Not applicable, because no patient is classified as positive for the outcome.

Supplementary Figure 1 shows the predicted probabilities of 30-day mortality by mortality status. The model calibration suggests a reasonably good fit for the model (Supplementary Fig. 2, likelihood-ratio statistic: 2.81, P = 0.094), which provides accurate predictions for almost the entire range of the death probability. The predicted probabilities stay close to the ideal calibration line for low and high probabilities of death. Sensitivity analyses showed no association between comorbidities and 30-day mortality or interaction between comorbidities and age; no association between systemic treatments, including chemotherapy, and null increase in predictive performance was observed, regardless of whether comorbidity was included in the model. Additionally, in sensitivity analysis we assessed whether our model was consistent to different windows of time (0–29, 0–35 and 0–45 days) and we applied our point score system to predict 3- and 6-month mortality in the same patient cohorts. We found similar values of NPV and PPV (Supplementary Tables 1 and 2).

## Discussion

We found that primary lung cancer, peripheral blood NLR, and plasma urea were strong predictors of short-term mortality among patients with stage IV cancer. Our score system was a good predictor of short-term mortality; performance metrics by ROC curves and calibration curves showed high model discrimination and calibration, respectively.

More recent and successful studies on predictive models for survival after palliative RT in patients with advanced cancer were conducted by Chow *et al*. and Kristnan *et al*.^22–24^. These studies are similar; however, ours has important advantages. We developed a scoring system that uses objective measurements (complete blood counts, liver and renal function tests within 180 days) to determine the 30-day mortality of patients receiving palliative RT. Furthermore, our data were obtained from routine practice; this increased the model’s clinical applicability, unlike those by Chow *et al*., whose Radiotherapy Rapid Response Programme was established with an aim to provide quick palliative RT for patients with terminal cancer mostly referred from medical oncologists and palliative care doctors^22,23^. Our model for prediction of 30-day mortality was developed based on a large population of unselected adults referred for palliative RT (5,795 patients versus 395 in Chow *et al*. and 862 in Krishnan *et al*.). The concordance (C)-statistic is a measure of goodness-of-fit for binary outcomes in a logistic regression model. It represents the probability that the predicted and observed outcomes are concordant for a randomly selected pair of patients in the predictive model^25^. The C-statistic for TEACHH model and Chow’s model based on 3 risk factors were 0.59 and 0.65 respectively^23,24^, while the AUC (equivalent to C-statistic) in our model was 0.81 which is better. The AUC was cross-validated which reduces the optimism bias of the other two and was internally validated to provide higher consistency in absence of an external validation. It is an easy-to-calculate tool for patients with metastatic cancer who were referred for palliative RT and who account for 20–40% of patients treated in radiation oncology departments^26–29^. Furthermore, given the mandatory status of death certification in Hong Kong and the automated nature of RT and vital status data collection, data on the dates of the first course of palliative RT and death were reliable. The referring clinician’s indication was included in our definition of palliative RT, which was better than merely using predefined dose and fractionation schedules. Our model performed reasonably well across a range of cancer types and other variables, despite lacking genetic data, cancer-specific biomarkers, or any detailed information beyond EMR. This emphasized that commonly available data in EMR contain important predictors to identify clinically relevant outcomes in patients with cancer under palliative care. Most of the inputs to the model are standard structured data components in EMR. The model’s algorithm could easily integrate into existing clinical management systems, importing the data directly from the EMR without specialized infrastructure. Additionally, implementing the tool can continuously and independently validate the predictive power from an ongoing prospective cohort. This is important to reflect the secular trend in cancer epidemiology changes, treatment variations, and referral patterns in an evolving real-world setting.

The model outperformed clinician estimates of survival to guide appropriate clinical judgment in treatment, resource allocation, and early palliative care referrals with advanced care planning^30^. The NPV exceeded 90% which means patients have very high chance of staying alive beyond 30 days if predicted so by the model. This could be a better standpoint to start dialogue with patients. Realistic and honest disclosure of prognosis can encourage shared decision-making between the patient and the care team, with which the patient can settle personal, family, and financial issues earlier, instead of embarking on another course of treatment based on inaccurate prognosis. However, after thorough discussions with the patient and family, if the patient still opts for RT despite reasonable chance of early mortality, we argue that hypofractionation is preferred to avoid a protracted course of RT near death, given the well-documented evidence for equivalent effects in a range of symptoms^31^.

Regarding the choice of covariates and development of the model, patients referred for palliative RT often received oncological treatment and blood work before; hence, we included commonly performed biochemical or hematological markers. Clinical experience has shown that patients with lung cancer generally die earlier than patients with other cancers, such as breast cancer^14^, and patients with certain sites of metastases (e.g., bone only) live longer than patients with others, such as brain and spinal metastases with cord compression^32–35^. Since no data were available on sites of metastatic diseases, we substituted with irradiation data. Hence, we included primary cancer site and irradiation site in the score determination. Age may influence not only recommendations for treatment but also prediction of remaining lifespan, analyzed in our model. Moreover, clinician estimates of survival were excluded because they were likely based on experience and training, poorly reproducible, and not commonly recorded in routine electronic database.

Our study had limitations. First, the prediction model was built on data from patients treated with RT and might not be accurate for untreated patients. Second, our procedure for categorizing the predictor variables may not identify the cutoff values with the best discriminating capacity. Third, some important prognostic factors may have been omitted. For example, data on performance status and patient quality of life evaluations using validated scales, or of their frailty status^36,37^, considered prognostic in previous studies, were not analyzed^22^. However, we introduced patient comorbidities as proxy for patient frailties. Fourth, palliative RT use was at the oncologists’ discretion in some cases when curative and palliative intent treatment could not be distinguished (e.g., patients having limited metastasis receiving higher dose RT for better local control). Finally, we considered the patients for first course palliative RT without considering the effects of subsequent RT courses and other treatments.

A prediction tool using EMR data, retrieved from routine clinical practice, can accurately predict short-term mortality among patients with advanced cancer starting radiotherapy. Such tool could facilitate shared decision-making among the patients, family, and medical care team. Additionally, it could help clinicians identify patients unlikely to benefit from RT beyond 30 days and those who may instead benefit from earlier palliative care referral and end-of-life planning. Machine learning techniques have the potential to improve clinical decision-making by identifying those at increased risk of poor mortality^38^. In 3 studies summarized by a systematic review, machine learning techniques are better than routine logistic regression in building model for mortality prediction in older and/or hospitalized adults, if enough data are obtained^38–41^. Future research is needed to incorporate machine learning techniques and to determine the generalizability and feasibility of the application of prediction tool in clinical settings.

## Supplementary information

Supplementary information.

## Data Availability

The datasets generated during and/or analyzed during the current study are available from the corresponding author on reasonable request.
