# Supplementary material for: Prediction model for short-term mortality after palliative radiotherapy for patients having advanced cancer: a cohort study from routine electronic medical data
Source: Sci Rep. 2020 Apr 1;10:5779. doi: 10.1038/s41598-020-62826-x (PMC7113237; doi:10.1038/s41598-020-62826-x)
Supplement: Supplementary file 1 — Supplementary information. [file 41598_2020_62826_MOESM1_ESM.pdf]

Title

Prediction model for short-term mortality after palliative radiotherapy for patients having advanced cancer: a cohort study from routine electronic medical data

Authors' names

Shing Fung Lee, Hollis Luk, Aray Wong, Chuk Kwan Ng, Frank Chi Sing Wong, Miguel Angel Luque-Fernandez

Appendix 1. Characteristics of the Predictors

Predictors related to the patient's characteristics

We retrieved data on patient's age, sex, socioeconomic status, comorbidities. Age at first course palliative radiotherapy (RT) was categorised into four age groups: <55, 55-64, 65-74, and ≥75 years. Socioeconomic status was categorised into a binary predictor, based on whether patients were unemployed and having government financial support. Comorbidities were classified based on the Charlson comorbidity index retrieved using methods proposed by the Deyo et al,[1] we also used the Royal College of Surgeons modified Charlson score which reduces the number of comorbidities to 12 (myocardial infarction, congestive heart failure, peripheral vascular disease, cerebrovascular disease, dementia, chronic obstructive pulmonary disease, rheumatic disease, liver disease, diabetes mellitus, hemiplegia/paraplegia, renal disease and human immunodeficiency virus infection and acquired immune deficiency syndrome), removing some categories such as peptic ulcer disease (because it is not considered a chronic disease anymore), and grouping diseases together (e.g., diabetes mellitus codes with or without complications are grouped into one category).[2] The modified score does not assign weights to comorbidities, and instead categorises the number of comorbidities in three different groups: 0, 1, and ≥2 as a multimorbidity indicator.

Predictors related to disease and treatment characteristics

Primary site of cancer were coded and grouped by similar International Classification of Diseases-9 codes. We recorded the use of preceding radical/adjuvant RT, and their sites, doses and fractionations respectively. Treatment intent was indicated by the treating oncologist. The RT sites were coded from free-text and were grouped according to tissue types and anatomical sites, including bone, brain, spine, chest, breast, abdomen, pelvis, skin and soft tissue. To ensure data quality, the codes were cross-checked by SFL, HL, and AW, any discrepancy was resolved by discussion. When multiple palliative RT courses for a patient were started on the same date, the one with the greatest dose-fractionation was chosen to represent the concurrent treatment, assuming that one was delivered for a more important clinical indication. For patients who had radical or adjuvant radiotherapy before their palliative RT, we calculated the treatment-free interval (TFI) from the date of the first radical/adjuvant RT fraction to the date of the first palliative RT fraction.[3] We chose a clinically relevant time point (6, 12, and 24 months) as a cut-off values for the TFI. We opted not to treat TFI as a continuous variable due to expected small numbers of patients with TFI and to aid clinical utility. The use of anti-cancer medications including chemotherapy, targeted therapy, hormonal therapy, immunotherapy, bone modifying agents and drugs for supportive care such as dexamethasone and granulocyte colony

stimulating factor within 365 days before first course palliative RT was dichotomized as present or absent. Blood test results within 180 days closest to the start of RT were available, these included complete blood count (total white cell count, absolute lymphocyte count, absolute neutrophil count, absolute monocyte count, platelet, haemoglobin, red cell distribution width, mean corpuscular volume, mean corpuscular haemoglobin concentration, mean corpuscular haemoglobin, haematocrit), liver function test (alanine transaminase, aspartate transaminase, alkaline phosphatase, bilirubin, albumin), renal function test (serum creatinine, urea, sodium and potassium levels), serum calcium level, lactate dehydrogenase, inflammatory markers (C-reactive protein, erythrocyte sedimentation rate), haemoglobin A1c, and tumour markers (carcinoembryonic antigen, carbohydrate associated antigen 19-9, alpha-fetoprotein and cancer antigen 125).

Predictors related to healthcare provision factors

We recorded the number of episodes of emergency admission and admission to intensive care unit within 100 days before first course palliative RT. The type of surgery patients had, if any, was categorized as major, minor, or none.

Appendix 2. Steps to develop a point score system for predictor model [4]

The regression coefficients of the selected predictors were assigned integer scores. We organised the continuous predictors into categories and determine the midpoint for each category, then a reference category for each predictor (continuous, binary, and categorical) was chosen. For continuous variables, we determined how far each category is from the reference category, and then multiply each difference by the regression coefficient for that predictor to determine the difference in“regression units” (for binary and categorical variables, the “regression unit” was the regression coefficient for that predictor). Next, we defined one point in the points scoring system to be corresponding to one regression unit. The points (rounded to the nearest integer) associated with each of the categories of the predictors were determined. After determining the minimum and maximum possible points totals, we calculated the risk estimate for each points across the range by using the original model with the points scores (rounded) to get the predicted probabilities. The estimate was literally a new risk prediction model that approximated the full model.

Step 1: Organize the predictors into categories and determine the mid-point (MP) for each category

Step 2: Choose a reference category for each predictor (continuous, binary, and categorical), MP_{REF}

Step 3: Determine how far each category is from the reference category in regression units. Note that continuous variables were fitted as such and therefore have a single regression coefficient. For continuous variables, determine how far each category is from the reference category, and then multiply the difference from the reference category by the regression coefficient for that predictor to determine the difference in "regression units." For binary and categorical variables, the "regression unit" is simply the regression coefficient for that predictor. Regression coefficients for red cell distribution width resulted from linear combination with their respective squared terms.

Step 4: Define the number of regression units that will correspond to one point in the point scoring system

Clinician preference was for a scaling factor of 1 in log peripheral blood neutrophil-lymphocyte ratio. Therefore, the scaling factor, $B = 0.5355749$

Although the scaling factor is based on log peripheral blood neutrophil-lymphocyte ratio, the same scaling factor is then used for all predictors.

Step 5: Determine the points (rounded to the nearest integer) associated with each of the categories of the risk factors.

$\mathbf{Points} = \beta \times (MP - MP_{REF})/B$

**Predictors**	**Categories**	**Transformed values**	**Mid-Point (MP)**	**Regression coefficient (β)**	**β×(MP-MP$_{REF}$)**	**Points**
Log peripheral white blood cell count [a,b]	**0–1.9**	**0–6.7**	**0.95**	0.3331105	0	0
	>1.9–2.3	>6.7–10.0	2.1		0.38307708	1
	>2.3–2.9	>10.0–18.2	2.6		0.54963233	1
Log peripheral blood neutrophil-lymphocyte ratio [a,b]	**-2.3–1.1**	**0.1–3.0**	**-0.6**	0.5355749	0	0
	>1.1–1.6	>3.0–5.0	1.35		1.04437106	2
	>1.6–2.3	>5.0–10.0	1.95		1.36571600	3
	>2.3–3.3	>10.0–27.1	2.8		1.82095466	3
Log plasma urea [a,b]	**-0.2–1.4**	**0.8–4.1**	**0.6**	0.4359264	0	0
	>1.4–1.8	>4.1–6.0	1.6		0.4359264	1
	>1.8–2.4	>6.0–11.0	2.1		0.6538896	1
Log serum bilirubin [b]	**0–3.2**	**0–25**	**1.6**	0.3981555	0	0
	>3.2–4.1	>25–60	3.65		0.81621878	2
	>4.1–6.4	>60–602	5.25		1.45326758	3
Serum albumin (g/dl)	11–33	-	22	-0.1062371	2.49657185	5
	34–38		36		1.00925245	2
	**39–52**		**45.5**		0	0

Lactate dehydrogenase (IU/L) [a]	**74–347**	-	**210.5**	0.0002763	0	0
	348–798		573		0.10015875	0
	799–1,774		1,286.5		0.2972988	1
Red cell distribution (%) [a]	**11.2–13.3**	-	**12.25**	0.2636713	0	0
	>13.3–14.0		13.65		0.36913982	1
	>14.0–14.7		14.35		0.55370973	1
	>14.–15.8		14.9		0.69872895	1
	>15.8–21.8		18.8		1.72704702	3
Attendance to emergency room	**0 time**	-	**0**	0.3583926	0	0
	1 time		1		0.3583926	1
	≥2 times		2		0.7167852	1
Sites receiving palliative RT	Whole brain or spinal RT	-	1	0.3702062	0.3702062	1
	**Otherwise**		**0**		0	0
Primary lung cancer	Yes	-	1	0.550104	0.550104	1
	**No**		**0**		0	0

[a] Upper bound trimmed at 95% percentile.

[b] mid-point values were based on log-scale.

RT, radiotherapy

Step 6: Determine the minimum and maximum possible points total

Minimal score = 0 + 0 + 0 + 0 + 0 + 0 + 0 + 0 + 0 + 0 + 0 + 0 = 0

Maximal score = 1 + 3 + 1 + 3 + 5 + 1 + 3 + 1 + 1 + 1 = 20

Step 7: Attach a risk estimate to each point total

The formula:

$$\hat{p} \approx \frac{e^{((\text{Points total} \times \text{B}) + \sum_{j=1}^{q}(\beta\text{j} \times \text{MPREFj}) - \sum_{i=1}^{p} \beta i \bar{X} i)}}{1 + e^{((\text{Points total} \times \text{B}) + \sum_{j=1}^{q}(\beta\text{j} \times \text{MPREFj}) - \sum_{i=1}^{p} \beta i \bar{X} i)}}$$

where:
j=1,…q are continuous predictors within the model
i=1,…p are all predictors within the model
$\sum_{i=1}^{p} \beta i \bar{X} i$ is the sum of the regression coefficients multiplied by the mean of each risk factor (centring value).

$\sum_{j=1}^{q}(\beta\text{j} \times \text{MPREFj})$ = ($\beta_{\text{log white cell}}$×0.95) + ($\beta_{\text{logNLR}}$×-0.6) + ($\beta_{\text{logurea}}$×0.6) + ($\beta_{\text{logbilirubin}}$×1.6) + ($\beta_{\text{albumin}}$×45.5) + ($\beta_{\text{LDH}}$×210.5) + ($\beta_{\text{RDW}}$×12.25) = - 0.6519388

$\sum_{i=1}^{p} \beta i \bar{X} i$ = ($\beta_{\text{log white cell}}$×2.13) + ($\beta_{\text{logNLR}}$×1.74) + ($\beta_{\text{logurea}}$×1.65) + ($\beta_{\text{logbilirubin}}$×2.12) + ($\beta_{\text{albumin}}$×35) + ($\beta_{\text{LDH}}$×710) + ($\beta_{\text{RDW}}$×16.2) + ($\beta_{\text{emergency}}$×0.30) + ($\beta_{\text{site}}$×0.34) + ($\beta_{\text{lung}}$×0.40) = 4.407572959

Substituting each points total in the formula in step 7 generates the table seen in the manuscript.

References

1. Deyo RA, Cherkin DC, Ciol MA. Adapting a clinical comorbidity index for use with ICD-9-CM administrative databases. J Clin Epidemiol 1992;**45**:613-9.

2. Brusselaers N, Lagergren J. The Charlson Comorbidity Index in Registry-based Research. Methods Inf Med 2017;**56**:401-06.

3. Williams M, Woolf D, Dickson J, Hughes R, Maher J. Routine clinical data predict survival after palliative radiotherapy: an opportunity to improve end of life care. Clin Oncol (R Coll Radiol) 2013;**25**:668-73.

4. Bonnett LJ, Snell KIE, Collins GS, Riley RD. Guide to presenting clinical prediction models for use in clinical settings. BMJ 2019;**365**:l737.

Supplementary Figure 1. Histograms for distributions of the predicted probabilities of 30-day mortality by vital status (alive versus dead).

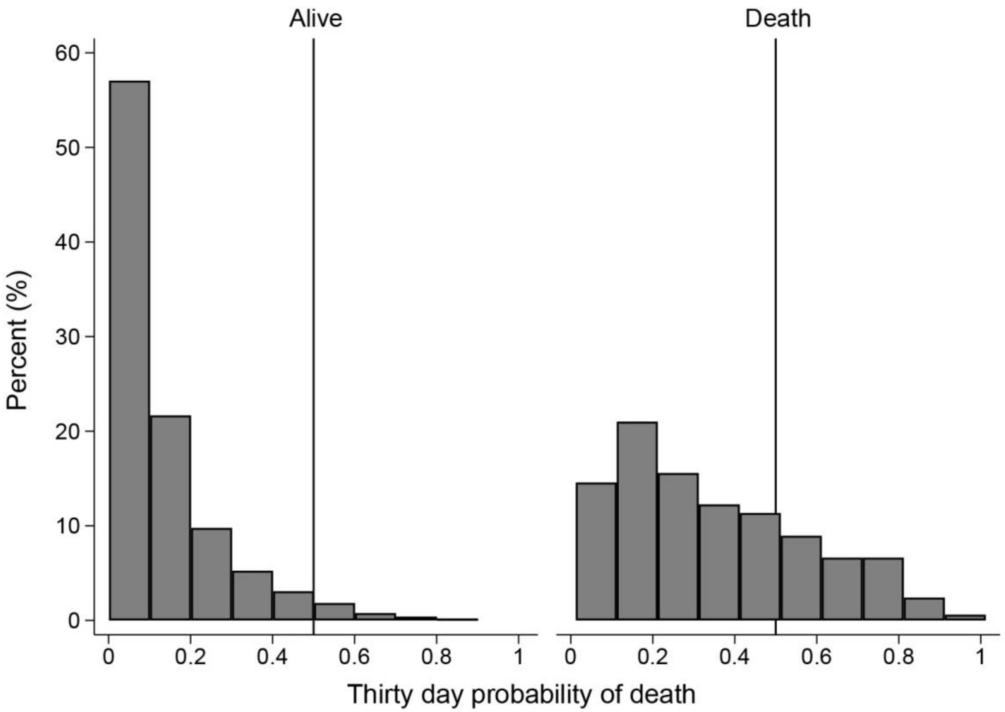

Supplementary Figure 2. Calibration curve for model 2 (likelihood-ratio statistic 2.81, P=0.094) and predictions for entire range of the death probability. It shows the fit of mortality data. The predicted probabilities must stay close to the ideal calibration line for low and high probabilities of 30 day mortality.

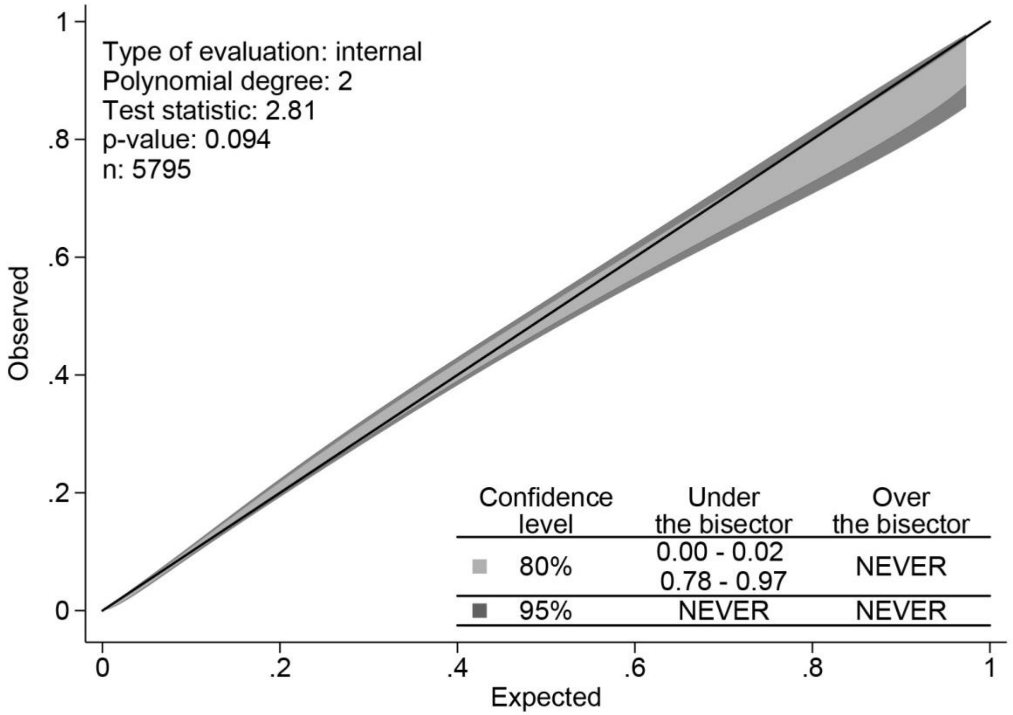

Supplementary Table 1A. performance for predicting 0 to 29-day mortality by the same chosen cut-off point score.

	**Death**	**Alive**	**Total**
**Test positive**	767	1,600	2,367
**Test negative**	201	3,227	3,428
**Total**	968	4,827	5,795

Sensitivity = 767 / 968 = 79.2%, specificity = 3,227 /4,827 = 66.9%, positive predictive value = 767 / 2,367 = 32.4%

negative predictive value = 3,227 / 3,428 = 94.1%

Supplementary Table 1B. performance for predicting 0 to 35-day mortality by the same chosen cut-off point score.

	**Death**	**Alive**	**Total**
**Test positive**	992	1,870	2,862
**Test negative**	172	2,761	2,933
**Total**	1,164	4,631	5,795

Sensitivity = 992 / 1,164 = 85.2%, specificity = 2,761 / 4,631 = 59.6%, positive predictive value = 992 / 2,862 = 34.7%

negative predictive value = 2,761 / 2,933 = 94.1%

Supplementary Table 1C. performance for predicting 0 to 45-day mortality by the same chosen cut-off point score.

	**Death**	**Alive**	**Total**
**Test positive**	1,294	2,297	3,591
**Test negative**	152	2,052	2,204
**Total**	1,446	4,349	5,795

Sensitivity = 1,294 / 1,446 = 89.5%, specificity = 2,052 / 4,349 = 47.2%, positive predictive value = 1,294 / 3,591 = 36.0%

negative predictive value = 2,052 / 2,204 = 93.1%

Supplementary Table 2. Probabilities of the outcome (3- and 6-month mortality) that correspond to the chosen cut-off point score.

Outcomes	Sensitivity in % (95% CI)	Specificity in % (95% CI)	Positive predictive value in % (95% CI)	Negative predictive value in % (95% CI)
3-month mortality	98.0 (97.3–98.5)	15.9 (14.7–17.1)	44.5 (43.2–45.8)	91.9 (89.4–93.8)
6-month mortality	99.9 (99.7–100.0)	1.1 (0.8–1.6)	58.5 (57.3–59.8)	90.0 (74.4–96.5)

Supplementary Table 3. Candidate predictors of 30-day mortality for palliative radiotherapy

Variables	Type of variables	Definitions
**Predictors related to the patient's characteristics**		
Sex	Binary	Male vs female
Age (years)	Continuous	
Socioeconomic status	Binary	government subsidy vs no
Comorbidities (Royal College of Surgeons modified Charlson score)	Categorical	scores 0, 1, ≥2
**Predictors related to disease and treatment characteristics**		
Primary cancer sites	Categorical	Head and neck, upper GI, lower GI, lung and thoracic, breast, soft tissue and skin, genitourinary, hematological, CNS, and others.
Preceding radical/adjuvant radiotherapy	Binary	Yes vs no
Current irradiated site		bone, brain, spine, chest, breast, abdomen, pelvis, skin and soft tissue.
Treatment-free interval	Categorical	Date of the first radical/adjuvant RT fraction to the date of the first palliative RT fraction (cut-off 6, 12, and 24 months)
[a]Use of chemotherapy within	Binary	Yes vs no

365 days before first course palliative RT		
[b]Use of targeted therapy within 365 days before first course palliative RT	Binary	Yes vs no
[c]Use of hormonal therapy within 365 days before first course palliative RT	Binary	Yes vs no
[d]Use of immunotherapy within 365 days before first course palliative RT	Binary	Yes vs no
[e]Use of bone modifying agents within 365 days before first course palliative RT	Binary	Yes vs no
[f]Use of drugs for supportive care within 365 days before first course palliative RT	Binary	Yes vs no
[g]Blood test results within 180 days before first course palliative RT	Continuous	Depending on particular blood tests
**Predictors related to healthcare provision factors**		
Admission to intensive care unit within 100 days before first course palliative RT	Continuous	Number of times of admissions
Visit to emergency room within 100 days before first course palliative RT	Continuous	Number of times of attendance
Previous surgery within 365 days before first course palliative RT	Categorical	Major vs minor vs no

Abbreviations: CNS, central nervous system; GI, gastrointestinal; RT, radiotherapy

[a] Chemotherapy: Cabazitaxel (prostate), capecitabine (multiple sites), carboplatin (multiple sites), Chlorambucil (lymphoma), Cisplatin, cyclophosphamide (breast), docetaxel (multiple sites), doxorubicin (breast, lymphoma), epirubicin (breast), etoposide (lung), Fludarabine (lymphoma), Fluorouracil (multiple sites), gemcitabine (multiple sites), ifosfamide (sarcoma), irinotecan (multiple sites), ixabepilone (multiple sites), lenalidomide (myeloma), lomustine (CNS) , melphalan (myeloma), methotrexate (breast, CNS, lymphoma), mitomycin (anus), ofatumumab (sarcoma), procarbazine (CNS), oxaliplatin (multiple sites), paclitaxel (multiple sites), pemetrexed (lung, mesothelioma),

temozolomide (CNS), thalidomide (myeloma), thiotepa (hematological), topotecan (ovary), vinblastine (lymphoma), vincristine (lymphoma, CNS), vinorelbine (breast, lung)

[b]Targeted therapy: Afatinib (lung), Alectinib (lung), Avelumab (multiple sites), Axitinib (renal cell carcinoma), Bevacizumab (multiple sites), Bortezumib (myeloma), certinib (lung), cetuximab (colon, head and neck), cobimetinib (melanoma), Crizotinib (lung), Dabrafenib (melanoma), erlotinib (lung), everolimus (renal cell carcinoma, breast), gefitinib (lung), imatinib (gastrointestinal stromal tumor, leukemia), lapatinib (breast), lenvatinib (hepatocellular carcinoma, thyroid), osimertinib (lung), palbociclib (breast), panitumumab (colorectal),pazopanib, ramucirumab (stomach), regorafenib (multiple sites), ribociclib (breast), rituximab (lymphoma), sorafenib (hepatocellular carcinoma), sunitinib (renal), temsirolimus (renal cell carcinoma), trametinib (melanoma), Trastuzumab (breast, stomach), vemurafenib (melanoma)

[c]Immunotherapy: Pembrolizumab (multiple sites), nivolumab (multiple sites), atezolizumab (multiple sites), Durvalumab (lung), ipilimumab

[d]Hormonal therapy: Abiraterone (prostate), Anastrozole (breast), Bicalutamide (prostate), degarelix (prostate), enzalutamide (prostate), flutamide (prostate), fulvestrant (breast), Goserelin (prostate, breast), letrozole (breast), Tamoxifen (Breast)

[e]Bone modifying agents: denosumab, pamidronate, zoledronate

[f]Drugs for supportive care: dexamethasone, filgrastim, ondansetron, prednisolone, nivestim

[g]Blood tests: complete blood count (total white cell count, absolute lymphocyte count, absolute neutrophil count, absolute monocyte count, platelet, haemoglobin, red cell distribution width, mean corpuscular volume, mean corpuscular haemoglobin concentration, mean corpuscular haemoglobin, haematocrit), liver function test (alanine transaminase, aspartate transaminase, alkaline phosphatase, bilirubin, albumin), renal function test (serum creatinine, urea, sodium and potassium levels), serum calcium level, lactate dehydrogenase, inflammatory markers (C-reactive protein, erythrocyte sedimentation rate), haemoglobin A1c, and tumour markers (carcinoembryonic antigen (CEA), carbohydrate associated antigen 19-9 (CA19-9), alpha-fetoprotein (AFP) and cancer antigen 125 (CA125))
